# 1-year outcomes of patients implanted with the Perceval sutureless valve: the Japanese post-marketing surveillance study

**DOI:** 10.1007/s00380-023-02240-1

**Published:** 2023-02-11

**Authors:** Hiroshi Niinami, Yoshiki Sawa, Tomoki Shimokawa, Satoru Domoto, Yoshitsugu Nakamura, Taichi Sakaguchi, Toshiaki Ito, Koichi Toda, Atsushi Amano, Borut Gersak

**Affiliations:** 1 Department of Cardiovascular Surgery, The Heart Institute of Japan, Tokyo Women’s Medical University Tokyo, Tokyo, Japan; 2 Cardiovascular Surgery, Osaka University, Suita, Japan; 3 Cardiovascular Surgery, Sakakibara Heart Institute Tokyo/Teikyo University, Fuchu, Japan; 4 Cardiovascular Surgery, Chiba-Nishi General Hospital, Matsudo, Japan; 5 Cardiovascular Surgery, The Sakakibara Heart Institute of Okayama, Okayama, Japan; 6 Cardiovascular Surgery, Japanese Red Cross Nagoya Daiichi Hospital, Nagoya, Japan; 7 Cardiovascular Surgery, Juntendo University Hospital, Tokyo, Japan; 8 Medical Faculty/Nisteri, Medicine and Research, University of Ljubljana, Phoenix, AZ USA

**Keywords:** Sutureless aortic valve, Clinical and hemodynamic outcomes, Japan

## Abstract

Sutureless offers an alternative to standard valves in surgical aortic valve replacement (SAVR). We sought to confirm the efficacy and safety of the Perceval sutureless valve in Japanese patients. Prospective observational study of 204 patients who underwent SAVR with Perceval at 19 sites in Japan between March and December 2019. The primary outcomes were 30-day mortality and postoperative complications; the secondary outcome was all-cause mortality at 1 year. Efficacy outcomes were changed in New York Heart Association (NYHA) class, pressure gradients, effective orifice area (EOA), EOA indexed to body surface area (EOAi) and severity of aortic regurgitation. Mean age was 77.7 years, 62.7% were female. Procedural success rate was 99.0%. The median cross-clamp and cardiopulmonary bypass times were 68.0 and 108 min. Perceval size *S* and *M* were implanted in 95 (46.6%) and in 76 (37.3%) of patients, respectively. The 30-day and late mortality rate were 0.5% and 4.4%, while the new permanent pacemaker implantation rate was 4.4%. Mean pressure gradient was 13.0 mmHg at discharge, reaching 11.0 mmHg at 1 year; while the mean EOA was 1.5 cm^2^ at discharge remaining stable up to 1 year. No moderate or severe leakages were present at discharge or at 1 year. NYHA class improved by ≥ 1 level in 55.1% of the patients at discharge and in 69.4% of the patients at 1 year. 1-year outcomes of SAVR with the Perceval sutureless valve in Japanese patients were favorable. This valve offers a promising alternative to conventional biological AVR in this Japanese population.

## Introduction

Surgical aortic valve replacement (SAVR) remains the gold standard treatment for those patients with isolated aortic valve stenosis (AS) not suitable for transcatheter aortic valve replacement (TAVR) as well as in a variety of other clinical settings, where TAVR alone will just treat AS, but the other pathologies like coronary artery disease, atrial fibrillation, mitral valve pathology will need surgical approach [1]. Rapid deployment and sutureless valves [2, 3], introduced more than 10 years ago, can be considered an important alternative to conventional valves used for SAVR either in isolated or concomitant procedure setting [4].

The prospective randomized PERSIST-AVR (Perceval Sutureless Implant Versus Standard-Aortic Valve Replacement) trial [5] showed that the Perceval sutureless valve reduced operative time and was non-inferior to conventional valves for MACCE (major adverse cerebral and cardiovascular events) at 1 year in patients with severe AS undergoing isolated or concomitant SAVR procedures. The CAVALIER (Safety and Effectiveness Study of Perceval *S* Valve for Extended CE Mark) study [6] had the longest 5 years follow-up data with sutureless technology in a prospective, multicenter study. The results of the study showed that Perceval sutureless valve was associated with low rates of death and morbidity, consistent with rates in large, randomized trials on transcatheter and surgical valves in similar risk populations and showed good hemodynamic performances. The PERSIST-AVR study was conducted in 12 countries in Europe, North America, Chile and Israel, whereas the CAVALIER study was conducted solely in Europe; no data are currently available for Japan. Compared with patients in Western countries, elderly Japanese patients tend to show smaller bodies and have smaller aortic annulus diameter [7, 8]. The purpose of this study was to evaluate the outcomes of SAVR with the Perceval sutureless valve in Japanese patients up to 1 year of follow-up.

## Materials and methods

### Study population

This is a prospective observational study of 204 consecutive patients with severe aortic stenosis or steno-insufficiency who underwent SAVR with the Perceval sutureless valve (Corcym Canada Corp, Burnaby, BC, Canada) at 19 sites in Japan between March and December 2019. Patients with valves smaller than 19 mm or bigger than 27 mm, pure aortic regurgitation, or type 0 bicuspid aortic valve were excluded. Up to our knowledge, this is the first prospective multicenter Japanese trial to evaluate the safety and performance of the Perceval sutureless valve.

The Perceval sutureless valve (Perceval) is a sutureless aortic bioprosthetic that gained European Conformity (CE) mark approval in 2011. Details about the Perceval valve have been previously described [9]. Briefly, the biological component of the Perceval valve consists of glutaraldehyde-fixed bovine pericardium treated with homocysteic acid to mitigate calcification. The stent comprises an elastic nickel-titanium alloy covered by Carbofilm to improve biocompatibility. The stent supports the valve and holds it in place without the need for a permanent suture. The valve is collapsed with a proprietary accessory device that reduces the diameter of the prosthesis without damaging the prosthesis leaflets. The Perceval valve is available in four sizes covering annuli from 19 to 27 mm (*S*: 19–21 mm, *M*: 21–23 mm, *L*: 23–25 mm, *XL*: 25–27 mm).

Proctoring on the Perceval valve implant, led by experienced implanters, was done for all sites at the start of the study. In Japan, each surgeon required proctoring for a minimum of 5 cases (compared with 2 cases in other countries) before performing surgery independently. Additionally, during the proctorship training, specific attention was given to the sizing technique, highlighting the importance to not oversize the Perceval valve.

### Surgical technique

A full sternotomy or minimally invasive cardiac surgery approach was chosen according to the surgeon’s preference, after performing their third case (in the initial three cases full sternotomy was the mandatory approach). Transverse aortotomy was performed approximately 3.5 cm above the annulus in all patients after establishment of cardiopulmonary bypass (CPB). In more than half of the patients, the Cavitron Ultrasonic Surgical Aspirator (CUSA) was used on aortic annulus, after the calcified aortic leaflets were removed by scissors, to maximally decalcify the landing site for inflow subannular part of the Perceval valve.

After complete decalcification of the annulus, 3 guiding sutures were placed 2 mm below each nadir of the annulus. We determined the valve size by measuring the aortic annulus using manufacturer-specific annular valve sizers. Specific attention was given not to oversize the valve [10]. Once the valve was placed, it was dilated with a balloon catheter for 30 seconds at 4 atmospheres with warm saline solution irrigation, then the guiding sutures were removed. After weaning from CPB, intraoperative transesophageal echocardiography was performed to confirm the correct positioning of the valve and to assess for the presence of a paravalvular leak (PVL).

### Data collection and follow-up

Preoperative patient data were entered into dedicated case reports forms. All the patients received prior to the operation a CT scan according to the individual hospital practice and the anatomical characteristics important for Perceval valve implantation were measured (annular size, sino tubular size, aortic valve morphology). Intraoperative data included the aortic valve disease, surgical approach, morphology of aortic valve, perioperative transthoracic or transesophageal echocardiographic results, information on the implant device, associated procedures, and procedure time.

Follow-up data (clinical findings, echocardiographic data, antithrombotic or antiplatelet medications, adverse events, and device deficiencies) were collected at discharge from the hospital, at 6 months and 1 year, and annually to 5 years after implantation. This report describes the clinical outcomes up to 1 year.

### Definitions and study outcomes

Procedural success was defined as device implantation without severe paravalvular regurgitation just after surgery or the need to convert to a conventional bioprosthesis. The primary outcomes were 30-day mortality and postoperative complications, and the secondary outcome was all-cause mortality during 1-year follow-up. Efficacy outcomes were the change in NYHA class and hemodynamic data such as mean pressure gradients, EOA, EOAi and severity of aortic regurgitation.

### Statistical analysis

Formal sample size was not calculated for the study, but the number of patients to be included was mandated by the Japanese regulatory authority.

Categorical variables are presented as counts (%) and continuous variables as mean (standard deviation [SD]) or median (quartile 1, quartile 3) as appropriate. All statistical analyses were performed with SAS software (version 9.4; SAS Institute Inc). Perioperative events were defined as those occurring on the day of implant, while early and late events were the ones occurring ≤ 30 days or > 30 days post implant, respectively.

## Results

Patient baseline characteristics are shown in Table 1. Mean age of the population was 77.7 ± 5.3 years (94.1% were ≥ 70 years) and 62.7% were female. Mean body surface area (BSA) was 1.52 ± 0.20 m^2^. The mean Society of Thoracic Surgeons (STS) score and EuroSCORE II-predicted mortality rates were 5.2 ± 3.9% and 4.3 ± 5.7%, respectively. The most prevalent coexisting conditions were coronary artery disease (30.9%), heart failure (28.9%), and atrial fibrillation (15.7%). Fifty-nine patients (28.9%) had previously undergone cardiac interventions. Most of the patients (96.6%) were undergoing AVR for degenerative disease.

**Table 1:** Patient baseline characteristics (*N* = 204)

Variables	*N* (%) or mean ± SD (range) or median [*Q*1, *Q*3]
Age (years)	77.7 ± 5.3 (59–94)
Patients ≥ 70 years	192 (94.1)
Patients ≥ 80 years	69 (33.8)
Female sex	128 (62.7)
Body surface area	1.5 ± 0.2
Body mass index (kg/m^2^)	22.9 ± 3.6
EuroScore II	4.3 ± 5.7
STS score	5.2 ± 3.9
Primary cause of disease	
Degenerative	197 (96.6)
Chronic rheumatic	4 (2.0)
Other	3 (1.5)
Coexisting conditions	
Coronary artery disease	63 (30.9)
Heart failure	59 (28.9)
Myocardial infarction	10 (4.9)
Pulmonary hypertension	8 (3.9)
Stroke	11 (5.4)
Arrythmia	
Atrial fibrillation	32 (15.7)
Paroxysmal atrial fibrillation	11 (5.4)
Bundle branch block	12 (5.9)
Paced rhythm	4 (2.0)
Sustained ventricular fibrillation	1 (0.5)
History of cardiac intervention	35 (17.2)
Coronary artery bypass graft surgery	1 (0.5)
Percutaneous coronary intervention	24 (11.8)
Pacemaker implantation	4 (2.0)
Valve replacement	4 (2.0)
Aortic valve	3 (1.5)
Mitral valve	1 (0.5)
Other	2 (1.0)

*Q1/Q3* quartile, *SD* standard deviation, *STS* Society of Thoracic Surgery

Procedural data are shown in Table 2. Three-quarters of the patients (74.5%) underwent a full sternotomy, 46.6% had concomitant procedures, and 26.0% had CABG. Twenty patients (9.8%) presented with a bicuspid valve. Overall, 46.6% of patients were implanted with a size *S* valve (19−21 mm) and 37.3% with a size *M* (21−23 mm) valve. Median pump time was 108.0 [82.5–147.3] min and cross-clamp time was 68.0 [54.0–96.5] min. Procedural success was achieved in 99.0% (202/204) of patients. In one patient implanted with an *XL* valve, the device was removed after aortic cross-clamp removal due to its suprannular migration, which resulted in severe PVL. The reason for this migration was the wrinkling of the aortic wall in Valsalva sinuses, which was noted after the second cross-clamp inspection. In fact, the combination of thin aortic walls, typical of Japanese patients, and an excessive traction on guiding sutures during implantation pulls the aortic annulus upward and creates wrinkles. These wrinkles to our knowledge are not uncommon in Japanese patients with thin, pliable and noncalcified aortic walls, even though we believe this can happen also to patients of different nationalities.

**Table 2:** Intraoperative data (*N* = 204)

Variable	*N* (%) or median [*Q1*, *Q3*]
Surgical approach	
Median sternotomy	152 (74.5)
Minithoracotomy	51 (25.0)
Ministernotomy	1 (0.5)
Aortic valve	
Previous prosthesis	3 (1.5)
Bicuspid	20 (9.8)
Tricuspid	181 (88.7)
CUSA usage rate	131 (64.2)
Number of valves attempted for implantation	
1	195 (95.6)
2	9 (4.4)
Implant success overall	202 (99.0%)
Implant failure	
*S*	1 (0.5)
*XL*	1 (0.5)
Valve size	
*S*	95 (46.6)
*M*	76 (37.3)
*L*	26 (12.7)
*XL*	5 (2.5)
Concomitant cardiac procedures	95 (46.6)
CABG	53 (26.0)
Left atrial appendicectomy	26 (12.7)
Mitral valve repair or replacement	9 (4.4)
Tricuspid valve repair	3 (1.5)
Mitral valve repair or replacement and tricuspid valve repair	9 (4.4)
Myectomy	6 (2.9)
MAZE/Pulmonary vain isolation	8 (3.9)
PCI	2 (1.0)
Pacemaker implant	1 (0.5)
Other	2 (1.0)
Procedure time	
All patients	
Pump time (min)	108.0 [82.8, 147.3]
Cross-clamp time (min)	68.0 [54.0, 96.5]
Full sternotomy	
Pump time (min)	114.0 [82.8, 155.0]
Cross-clamp time (min)	69.0 [54.0, 102.0]
Minimally invasive cardiac surgery	
Pump time (min)	100.0 [83.5, 131.3]
Cross-clamp time (min)	65.0 [55.5, 82.0]
Isolated procedure	
Pump time (min)	93.0 [77.0, 116.0]
Cross-clamp time (min)	58.5 [51.8, 75.3]
Concomitant procedure	
Pump time (min)	136.0 [87.9, 193.9]
Cross-clamp time (min)	84.0 [50.3, 138.9]

*CABG* coronary artery bypass graft surgery, *PCI* percutaneous coronary intervention, *CUSA* Cavitron Ultrasonic Surgical Aspirator, *Q1/Q3* quartile, *SD* standard deviation

In the second patient, implanted with an *S* valve, procedural failure was due to stent infolding related to valve oversizing, resulting in moderate-to-severe PVL. Both patients underwent conversion to conventional valves. All the patients were on antiplatelet or antithrombotic treatment for 3 months after surgery, according to the Japanese guidelines.

At hospital discharge, no moderate or severe leakages were identified, and same results have been reported at 1 year, and during the 6 month follow-up.

**Fig. 1:**
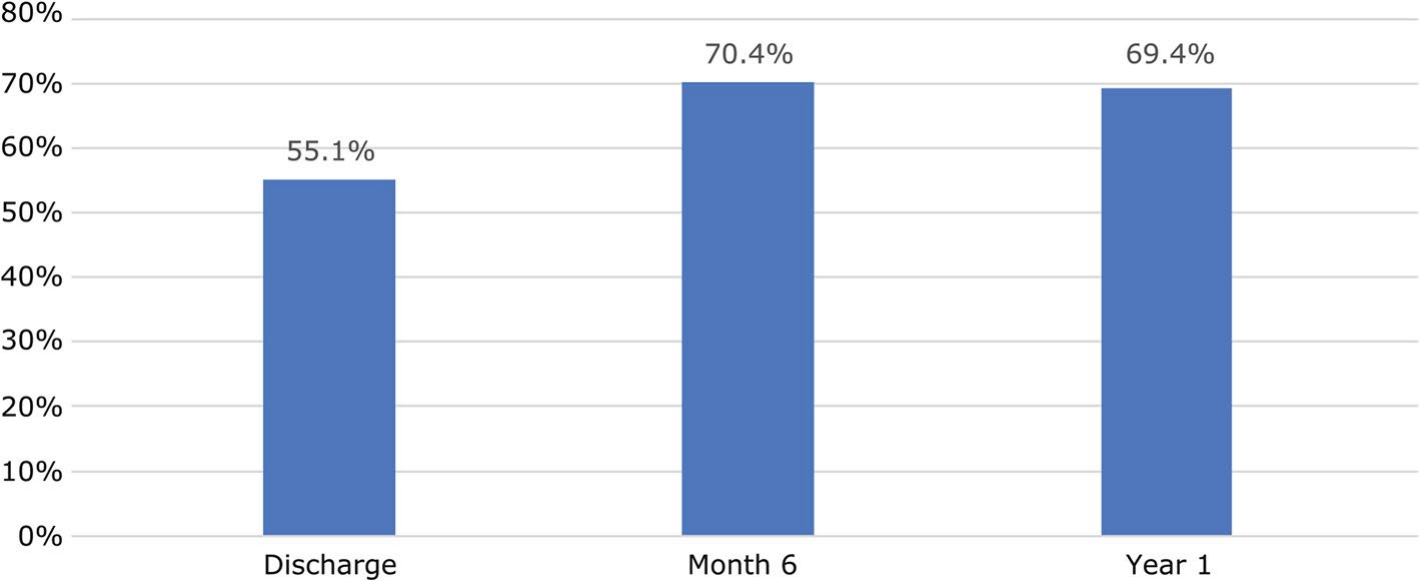
Percentage of patients with an improved NYHA functional class at by at least one level from baseline

**Fig. 2:**
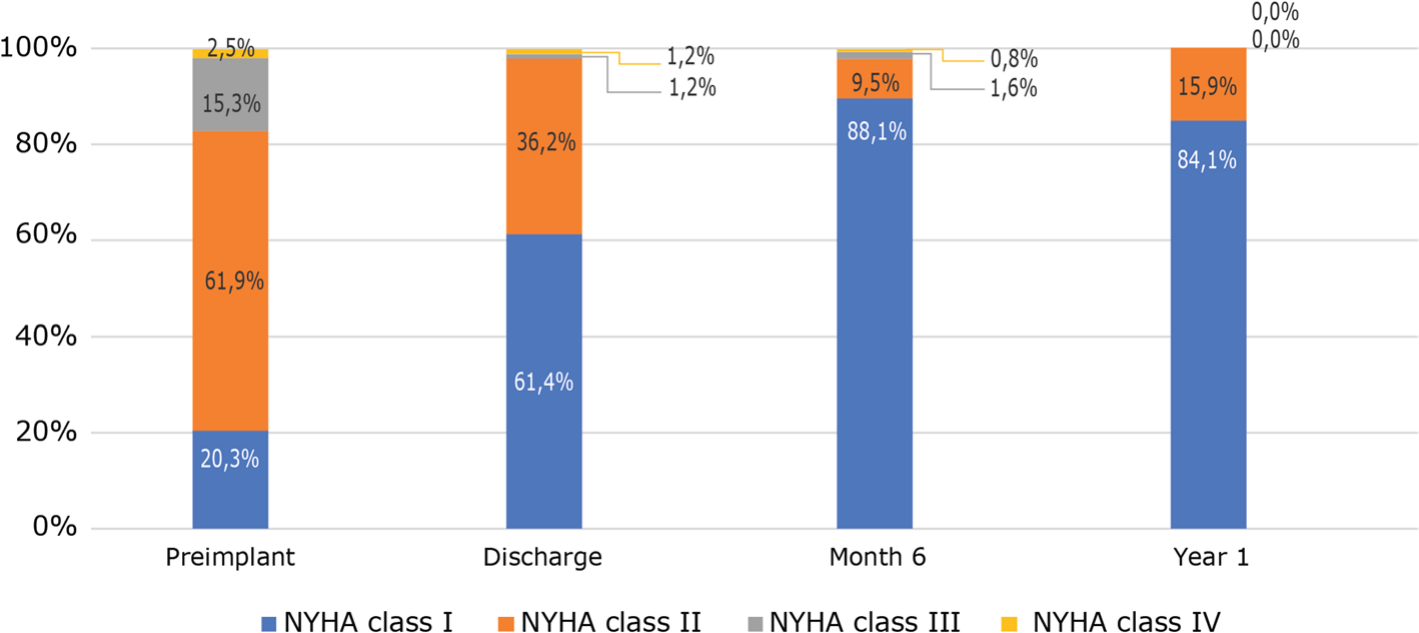
NYHA functional class improvement from baseline to 1 year

**Fig. 3:**
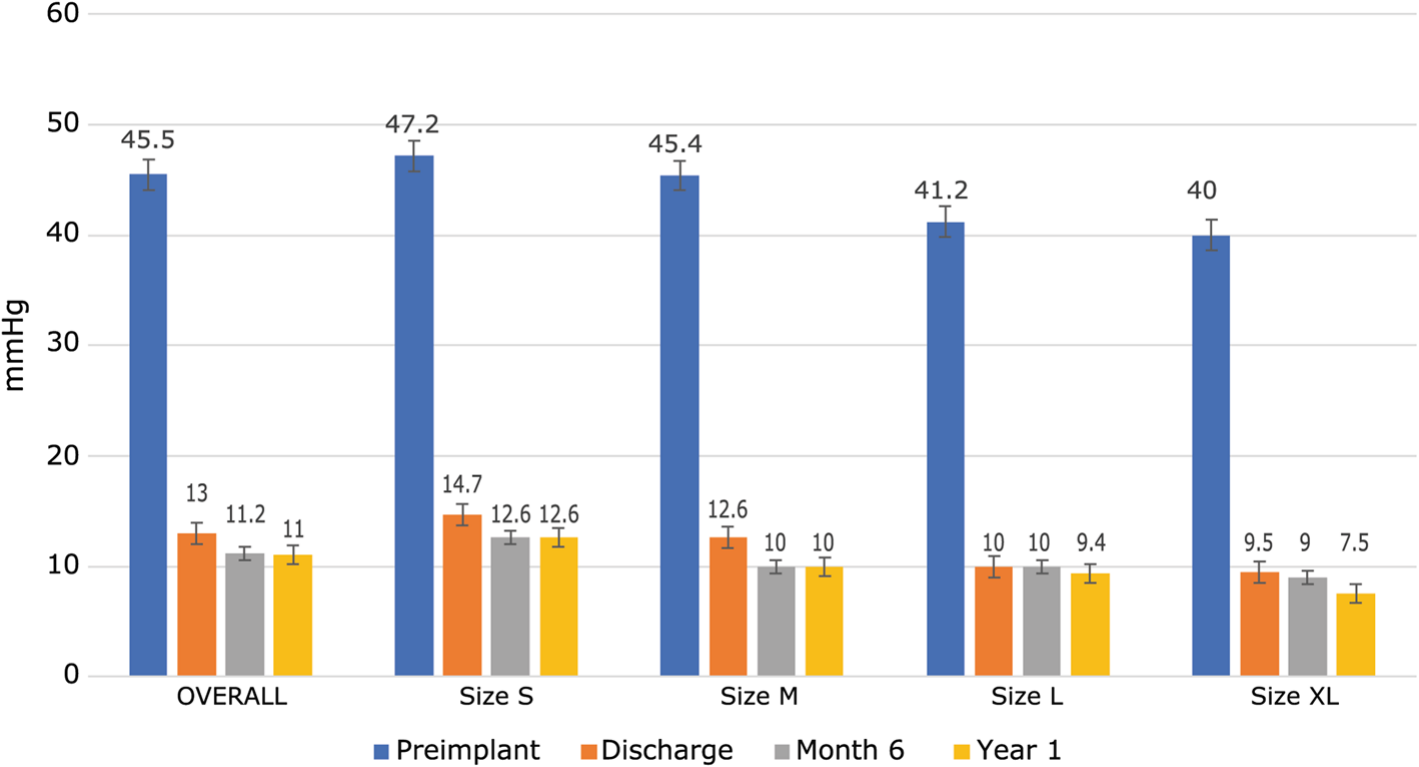
Mean pressure gradient by valve size and visit

**Fig. 4:**
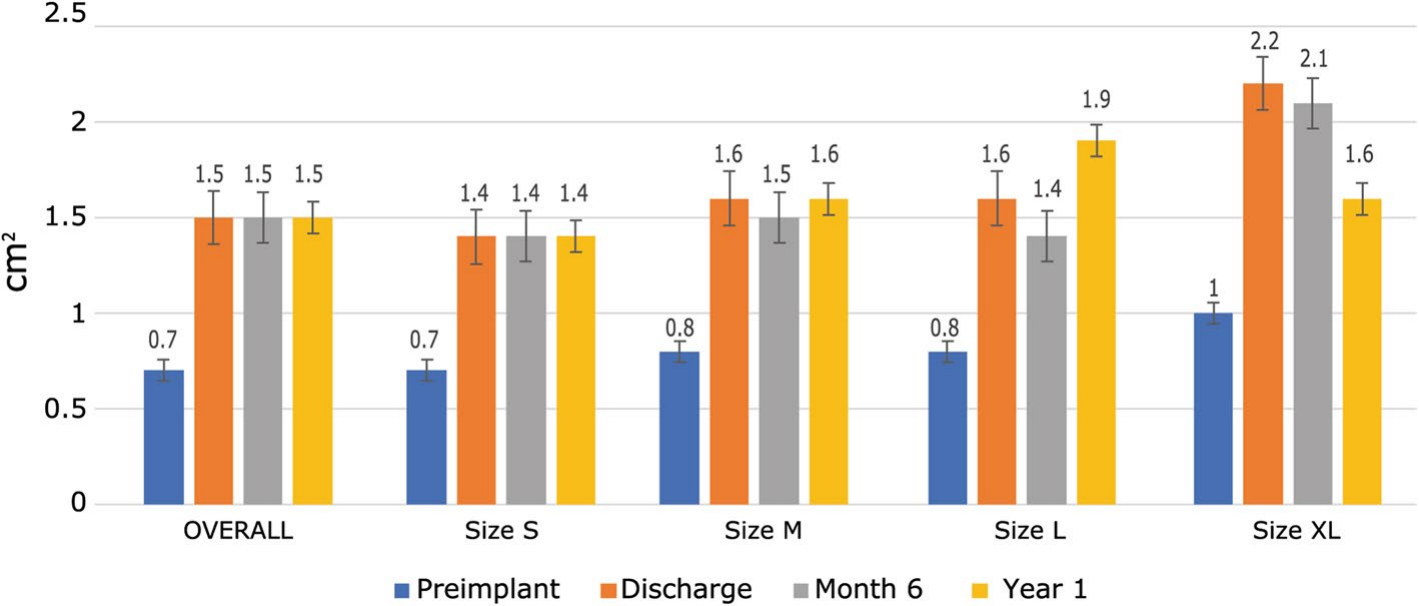
EOA by valve size and visit

NYHA functional class at discharge improved by at least one level in 91 patients (55.1%) (Fig. 1) and remained stable during follow-up:17.8% were in class III or IV preimplantation versus 2.4% at discharge and 0% at 1 year (Fig. 2). Mean pressure gradient decreased from 45.5 to 13.0 mmHg and reached 11.0 mmHg by 1 year (Fig. 3). Mean EOA increased from 0.7 cm^2^ before surgery to 1.5 cm^2^ at discharge and remained largely unchanged at 1 year (Fig. 4). Mean left ventricular ejection fraction improved from 59.8% at preimplantation to 64.4% at 1 year.

### Adverse events

Adverse events are detailed in Table 3. The 30-day mortality rate was 0.5% (1 patient developed sepsis and died 16 days after implantation). The late mortality was 4.4%.

**Table 3:** Adverse events: perioperative, early and late

Adverse event	Perioperative	Early (≤ 30 days)	Late (> 30 days)
	No. patients (%)		
Death		1 (0.5)	9 (4.4)
Complete atrioventricular block	5 (2.4)	3 (1.5)	
II degree atrioventricular block	1 (0.5)		
Valve thrombosis		1 (0.5)	
Myocardial infarction	1 (0.5)		
Procedural haemorrhage	1 (0.5)		
Platelet count decreased		1 (0.5)	
Device dislocation	1 (0.5)		
Aortic regurgitation – moderate/severe	0	0	0

**Fig. 5:**
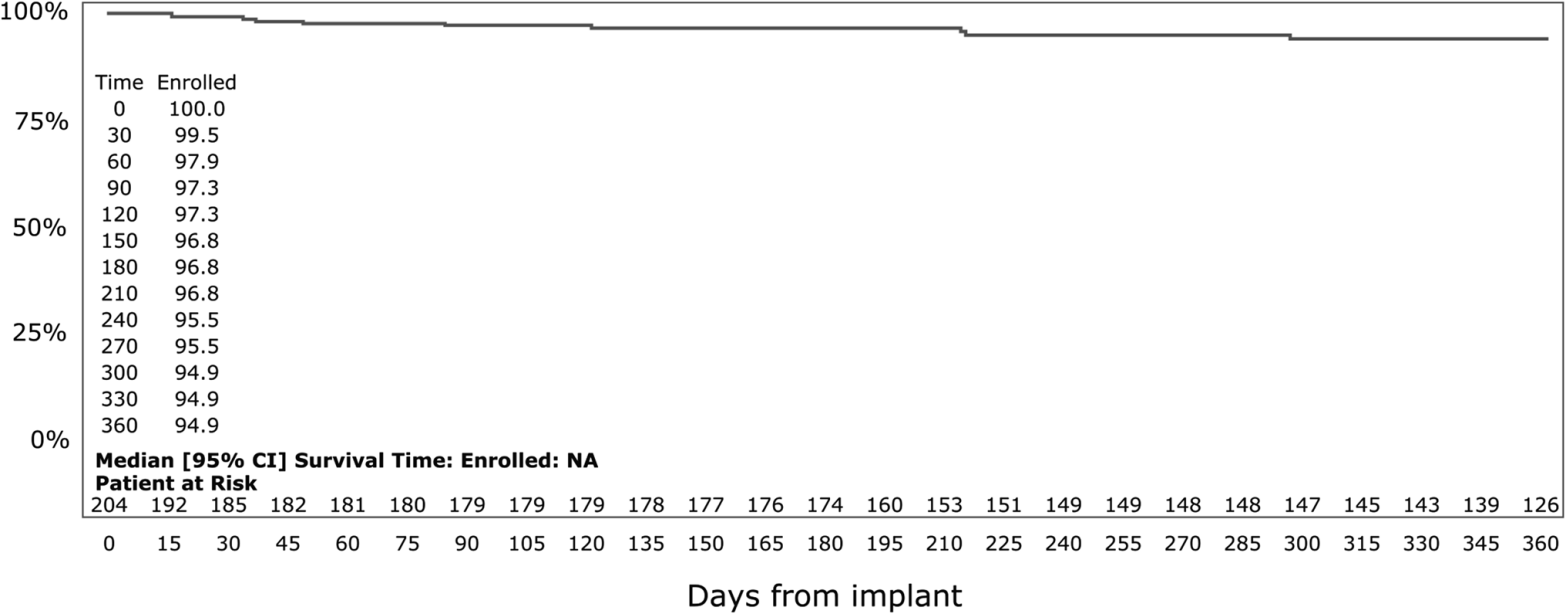
Kaplan–Meier survival

One patient underwent reoperation for valve thrombus at 19 days and died 36 days after implantation. Eight additional patients died after implantation during follow-up: 1 patient diagnosed with ileus and pneumonia died 33 days after implantation; 1 patient diagnosed with end-stage lung cancer died 122 days after implantation; 1 patient died for brain infarction after 85 days, 1 patient died for aspiration pneumonia after 49 days while 4 patients died of an unknown reason which was not related to the procedure or device. The Kaplan-Meier survival curve to 12 months is shown in Fig. 5.

New onset of the complete atrioventricular block was reported in 8 patients and second-degree atrioventricular block in 1 patient. All 9 patients (4.4%) underwent permanent pacemaker implantation, two of whom had been diagnosed with pre-operative conduction disturbances at baseline. In three cases oversizing was identified as the leading cause of the atrioventricular block, bicuspid type I was present in four cases, while in one patient there was an extensive decalcification of the left ventricular outflow tract due to calcification of the mitral-aortic continuity. Among the 9 patients, 2 have been implanted with Perceval valve size *S*, 3 with size *M*, 3 with size *L* and 1 with *XL* size.

## Discussion

The primary findings in this initial Japanese clinical experience were that (1) 1-year outcomes of SAVR with the Perceval valve in Japanese patients were favorable, also in patients with small anatomy. (2) Low PPI rate and no moderate or severe PVL were achieved by the complete decalcification using CUSA and by the selection of the proper sized valve.

Sutureless valves represent an innovative approach for SAVR, allowing for faster implantation compared with sutured and conventional valves, thus reducing cardiopulmonary and cross-clamp times [5, 11, 12]. This reduction in ischemic time is advantageous for all patients regardless of their risk profile [11, 13–15]. In our cohort, the cardiopulmonary bypass time and cross-clamp time can be perceived as longer than expected, even for isolated procedures, but this can be justified considering that 50% of the procedures have been done in RAT approach. In addition, Perceval valve is an ideal valve for minimally invasive approaches, being fully collapsible and implanted without the need for sutures, thus facilitating AVR through a small incision. Two recent studies have reported good outcomes following Perceval valve implantation with a minimally invasive approach [16, 18]. The possibility to reduce procedural time in the event of concomitant cardiac procedures can be advantageous for patients. In our cohort, almost half of the patients had to undergo AVR with concomitant procedure, with good mortality and morbidity outcomes, confirming previously published experience [16–20].

Few reports have been published about Perceval valve implantation in Asian populations despite in other countries including Thailand, Taiwan, and Korea and no data are available for Japan. Compared with patients in Western countries, elderly Japanese patients tend to show smaller bodies and have smaller aortic annulus diameter. In fact, the mean BSA was 1.52 ± 0.20 m^2^ in this study and this is smaller than any other study. 84% of the implanted valves were *S* or *M* size, therefore, addressing annuli with a diameter < 23 mm.

A premarketing study has been started in China recently (PERCEVAL *S* Valve Clinical Study for Chinese Registration [PERFECT] NCT03481387). The largest available published cohort is from Korea, with 121 patients (mean age 74.7 ± 6.2 years, 53.7% women) implanted between 2014 and 2019 [21]. In this Korean cohort, Perceval valve implantation provided a significant cardiac-related survival benefit with good early hemodynamic and clinical outcomes. Postoperative permanent pacemaker implantation was required in 9 patients (7.4%) because of new-onset arrhythmia, including atrioventricular block. However, after modifying the placement of the guiding suture, avoiding placement too deep, the rate of permanent pacemaker implantation decreased from 9.9% to 2.5%, but with no statistically significant difference between subgroups (*P* = 0.15).

The patients in our study were at higher risk than those in the Korean study (EuroScore II 4.3 ± 5.7% vs. 3.0 ± 4.3%, respectively) and almost half were undergoing concomitant cardiac procedures in both studies (46.6% vs. 45.5%), but the rate of complications in our cohort was low in comparison (e.g. in-hospital death 1.7% in the Korean study vs death ≤ 30 days 0.5% in our study, and atrioventricular block 7.4% vs 4.4%, respectively). The low rates of complications may reflect the correct sizing and positioning of the valve in this Japanese population, which may be due to several factors. First, sites experienced in cardiovascular were specifically chosen for this study, although low-volume centers also participated. The proctoring approach followed in Japan was extensive, with 5 cases being proctored versus the usual 2. Additionally, proctors scrubbed in and assisted with the procedure, providing ‘hands-on’ guidance, and emphasis was placed on correct sizing and positioning. The technique of the sizing used was described previously [22] where it has been clearly shown that even slight oversizing can lead to higher pacemaker implantations rates. A team-based approach was adopted where two surgeons were proctored in each center, rather than proctoring a single surgeon at each site. Patients were prescreened meticulously 1−2 weeks before surgery through a CT scan examination, and only those considered good candidates for the Perceval valve underwent the procedure. Patients with valves smaller than 19 mm or bigger than 27 mm, bicuspid aortic valve type 0, for example, were not considered suitable candidates for implantation. This approach likely led to low rates of PVL and permanent pacemaker implantation. No moderate or severe PVL was reported at the postoperative visit or at 1 year, confirming that a perfect seal can be achieved at the annulus when the valve is implanted correctly. We believe that complete decalcification can prevent the PVL by improving the fitting between the Perceval valve and the aortic annulus and can reduce the stress on the conduction system by equalizing the stress on the aortic annulus.

In our Japanese study, 83.9% of the patients received a small or medium size valve versus 62.0% of patients in the Korean study [19] and 40.4% in the multinational PERSIST-AVR trial [5]. The rate of permanent pacemaker implantation was 4.4% in our study, versus 9.9% (at the start of the study) in the Korean study, decreasing to 2.5% after modifying the guiding suture placement, and 10.6% in the PERSIST-AVR trial. Consequently, in our study, the rate of permanent pacemaker implantation seems to be lower than in other large cohort studies due to the implementation of a comprehensive support from experienced proctors, the careful patient screening by computed tomography, the use of CUSA, the avoidance of valve oversizing and the attention to not position the valve too deep.

### Limitations

This is an observational study subject to the limitations of such studies, including the lack of a comparison group. The study included only Japanese patients and the findings may not be representative of other populations.

### Conclusions

1-year outcomes of SAVR with the Perceval valve in Japanese patients were favorable even in patients with small anatomy. The Perceval valve offers a promising alternative to conventional biological AVR in this Japanese population.

## Data Availability

The data underlying this article will be shared on reasonable request to the corresponding author.
